# Leukocyte and Platelet Characteristics of the Giant Panda (*Ailuropoda melanoleuca*): Morphological, Cytochemical, and Ultrastructural Features

**DOI:** 10.3389/fvets.2020.00156

**Published:** 2020-03-24

**Authors:** Spencer P. Kehoe, Nicole I. Stacy, Salvatore Frasca, Tracy Stokol, Chengdong Wang, Kathryn Strayer Leach, Li Luo, Sam Rivera

**Affiliations:** ^1^Department of Veterinary Services, Zoo Atlanta, Atlanta, GA, United States; ^2^Department of Comparative, Diagnostic, and Population Medicine, College of Veterinary Medicine, University of Florida, Gainesville, FL, United States; ^3^Department of Population Medicine and Diagnostic Sciences, College of Veterinary Medicine, Cornell University, Ithaca, NY, United States; ^4^Chengdu Research Base of Giant Panda Breeding, Northern Suburb Chengdu, Sichuan, China

**Keywords:** *Ailuropoda melanoleuca*, cytochemistry, giant panda, hematology, immunocytochemistry, leukocyte morphology, transmission electron microscopy, *Ursidae*

## Abstract

The giant panda (*Ailuropoda melanoleuca*) is a vulnerable species and a charismatic member of zoological collections worldwide. Despite its importance as a representative species for global wildlife conservation efforts, no studies to date have described normal cell morphology or cytoplasmic constituents by traditional techniques such as cytochemical staining and evaluation of ultrastructural features. The objective of this study was to accurately identify and characterize the leukocytes and platelets of clinically healthy giant pandas using routine Wright-Giemsa stain, eight cytochemical stains, immunocytochemistry (CD3), and transmission electron microscopy (TEM) to further the collective understanding of normal cellular morphological features, cytochemical reactivity, and cytoplasmic contents found in health. Voluntary venipuncture was performed on four healthy individual animals (two adults and two juveniles), as part of routine preventive health evaluation. Blood was collected for routine and cytochemical stains, and into 2.5% glutaraldehyde for TEM. On Wright-Giemsa-stained blood films, leukocytes were differentiated into granulocytes (neutrophils, eosinophils, basophils) and mononuclear cells (lymphocytes, monocytes). Cytochemical staining revealed similar leukocyte and platelet staining patterns to those reported in other mammals, with some notable differences. By TEM, leukocytes with nuclear and cytoplasmic features of mononuclear cells were readily differentiated from granulocytes, and platelets had similar ultrastructural features to those reported in other mammals. Neutrophils were the predominant cell type followed by lymphocytes, while basophils were rare. Rare large or reactive lymphocytes, rare reactive monocytes, and rare large platelets were noted in apparently healthy giant pandas of this study. A unique mononuclear cell, with a moderately indented nucleus and shared cytochemical and ultrastructural characteristics of lymphocytes and monocytes, was discovered in this species. The combined cytochemical, immunocytochemical (CD3), and ultrastructural features of these unique cells more closely resemble those of monocytes, but the definitive cell lineage remains unknown at this time. This study provides novel information on giant panda leukocyte morphology and cellular constituents in health, shows the importance of manual blood film review, has important implications for hemogram interpretation in future clinical cases and research, and provides a baseline for future characterization and understanding of hemogram changes in response to disease.

## Introduction

The giant panda (*Ailuropoda melanoleuca*) is an endemic species to China, living in small populations isolated by fragmented landscapes and mountain ranges (1). Despite being an iconic animal and flagship species for global conservation efforts, the International Union for the Conservation of Nature's (IUCN) Red List recently downgraded the giant panda's status from endangered to vulnerable in 2016. Previously, the most prominent threat to the population was deforestation and consequent habitat loss. However, China's efforts to protect and restore the native forests where these animals live have been a major driving factor for the increasing population trend of giant pandas in the wild from ~1,596 in 2003 to 1,864 in 2016, and the subsequent reduction in their status (2–5). Despite no longer being classified as endangered, concern remains regarding their status as habitat fragmentation, population isolation, increasing livestock encroachment, infrastructure development, and tourism have emerged as new threats to the survival of the species (1, 2, 5–11). Of particular concern is the rapid rate of climate change and associated habitat changes, which may lead to additional loss of habitat by more than 50% according to some models (6, 8–10).

Given the continued concern for the conservation status of the giant panda, it is imperative to monitor these animals for infectious diseases of concern to the species and relevant environmental and anthropogenic risks. An essential requirement of any monitoring or disease identification program is knowledge of basic health parameters, such as behavior, morphometrics, physical findings, and results of clinical pathological and infectious disease testing. These parameters allow for the comprehensive evaluation of individuals or groups of animals under managed care or in the wild (12). One useful and readily available tool utilized for this purpose is hematological evaluation, as changes in hematological analytes can support the diagnosis of stress or various infectious or inflammatory conditions that might affect these animals.

Despite the importance of the giant panda, only two studies to date have provided limited descriptions of hematological findings and reference ranges (13, 14) in a low number of apparently healthy giant pandas (*n* = 17 and *n* = 7, respectively). However, neither of these studies provided a detailed description of normal white blood cell (WBC) morphological features or cytoplasmic constituents, as determined by cytochemical staining and electron microscopy. These techniques have been utilized successfully in various other non-domestic mammalian and non-mammalian species (15–17) to assist with accurate identification of cell lineages and detailed morphological characterization of blood cells, and to facilitate understanding of their functions. Correct leukocyte identification in turn allows for more accurate hemogram interpretation, which has clinical implications for the management and care of these animals. It is important to have baseline information on the morphological features, cytochemical staining characteristics, and cellular constituents of leukocytes in healthy pandas, so that deviations from the norm may be readily recognized. This will allow clinicians to better understand how the giant panda responds at the cellular level to disease, stress, and physiological conditions, such as growth, ageing, reproduction, and changes in habitat/diet in wild animals or husbandry in captive animals.

The purpose of this study was to describe and characterize leukocytes and platelets in the blood of clinically healthy captive giant pandas using Wright-Giemsa (a routine Romanowsky hematological stain), eight cytochemical stains, and transmission electron microscopy (TEM). With this data, we sought to establish baseline data for the typical morphological features and cytoplasmic constituents of leukocytes and platelets in the blood of healthy giant pandas. The information gained from this work will impact hemogram interpretation in future clinical cases and research, allow for more accurate hematological reference interval determination following the guidelines set forth by the American Society of Veterinary Clinical Pathology (18), and improve our understanding of leukocyte changes in response to disease, stress, and physiological changes in free-ranging and captive giant pandas.

## Materials and Methods

Ethics Statement: This study was carried out in accordance with all state, federal, international, and institutional guidelines, with an approved institutional animal care and use committee protocol (UF IACUC# 201706823).

**Figure 1:**
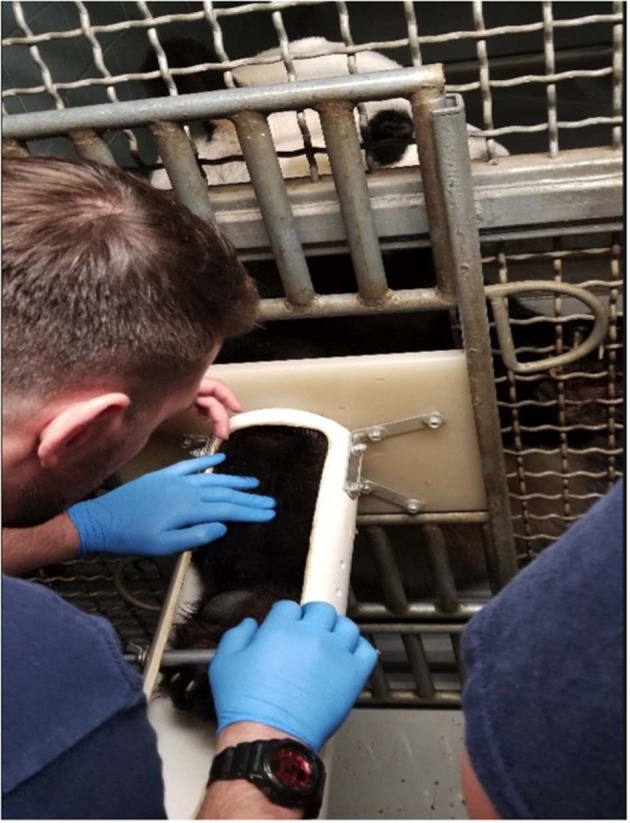
Modified phlebotomy sleeve made from polyvinyl chloride tubing and plastic housing, locked into the metal mesh of the enclosure, with the giant panda (*Ailuropoda melanoleuca*) reaching into the sleeve and grasping a horizontal metal bar. The patient remained in this position voluntarily, utilizing behavioral restraint and positive reinforcement while the antebrachium was clipped, alcohol was applied, and phlebotomy was performed.

Four clinically healthy giant pandas, consisting of one adult male and female and two juvenile females, were utilized for this study. All animals were housed at Zoo Atlanta, an Association of Zoos and Aquariums-accredited institution, which is one of only three institutions currently housing the eight giant pandas in the United States. Blood collection was performed as part of their annual preventive medical assessment, with all animals having been trained for phlebotomy while awake. Collection was accomplished via behavioral restraint, with each animal voluntarily placing their antebrachium into a modified phlebotomy sleeve made from polyvinyl chloride (PVC) tubing and grasping a horizontal bar (Figure 1). With the aid of positive reinforcement, they remained in position until shaving, alcohol preparation, and phlebotomy was complete. Blood was collected from the median antebrachial vein using a 22-gauge butterfly needle, and placed into a blood tube containing ethylenediamine tetraacetic acid (EDTA) for anticoagulation. All four animals were assessed as healthy based on a lack of reported or observed clinical abnormalities, and results of routine hematological and serum biochemical testing performed as part of their annual preventative health assessment, which were within previously published intervals for the species (13, 14).

Numerical results for hematological tests were obtained from the EDTA-anticoagulated blood with an automated Sysmex XN-10 series hematology analyzer (Sysmex America, Inc., Lincolnshire, IL). Ten standard wedge-type direct blood films were prepared from the blood and air-dried, and were used to assess leukocyte morphological characteristics via light microscopy using a Wright-Giemsa stain (Harleco®, EMD Millipore, Billerica, Massachusetts, USA) and cytochemical staining characteristics using eight stains. A board-certified clinical pathologist (NIS) performed the leukocyte differential by counting 200 WBC on two Wright-Giemsa stained blood films per animal (100 WBC per smear) and conversion to absolute numbers, as per standard laboratory practice (Table 1). Cytochemical staining for alkaline phosphatase (ALP), α-naphthyl butyrate esterase (ANBE), myeloperoxidase (MPx), chloroacetate esterase (CAE), and Sudan black (SBB) were performed using commercial kits (ALP, Procedure No. 85; ANBE, Procedure No. 181; MPx, Procedure No. 390; CAE, Procedure No. 91; SBB, Procedure No. 380; Sigma-Aldrich, St. Louis, MO, USA) as previously described (19), with blood from a horse (ALP and ANBE) and dog (MPx, CAE, and SBB) as positive controls. Periodic-Acid-Schiff (PAS), Toluidine blue (TB, pH 2.5–3.0), and Luna stains were performed using established protocols (20, 21), with histological sections of eosinophilic infiltrates, gastrointestinal tract, and a mast cell tumor used as positive controls. All cytochemical stains were performed at the Clinical Pathologic Laboratory in the Animal Health Diagnostic Center (AHDC) at Cornell University, and examined independently by two authors (TS and NS). The strength of the staining reaction (negative, equivocal, 1+ to 3+) and pattern of staining (diffuse in cytoplasm, granular, etc.) were subjectively graded. Only positive cytochemical reactions are provided in the text for ease of reading.

**Table 1:** Comparative hematology data from four healthy captive giant pandas (*Ailuropoda melanoleuca*), one adult male and female and two juvenile females, used in this study and housed at the same AZA-accredited institution.

**Hematology**	**Adult male**	**Adult female**	**Juvenile female #1**	**Juvenile female #2**
Hematocrit (%)	40	45	45	35
Hemoglobin (g/dL)	14.0	15.1	14.3	12.1
RBC (x10^12^/L)	6.52	7.24	7.28	5.93
MCV (fL)	61	62	62	59
MCH (pg)	21.5	20.9	19.6	20.4
MCHC (g/dL)	35.0	33.6	31.8	34.6
WBC x10^3^/uL	6.70	6.80	7.40	11.90
Band Neutrophils (x10^3^/uL)	0	0	0	0
Segmented Neutrophils (x10^3^/uL)	4.30	5.20	4.80	7.60
Lymphocytes (x10^3^/uL)	1.80	1.20	2.20	3.10
Monocytes (x10^3^/uL)	0.13	0.14	0.22	0.36
Unique mononuclear cells (x10^3^/uL)	0.20	0.07	0.15	0.48
Eosinophils (x10^3^/uL)	0.27	0.20	0.07	0.36
Basophils (x10^3^/uL)	0	0	0	0
Band Neutrophils (%)	0	0	0	0
Segmented Neutrophils (%)	64	76	65	64
Lymphocytes (%)	27	18	29	26
Monocytes (%)	2	2	3	3
Unique mononuclear cells (%)	3	1	2	4
Eosinophils (%)	4	3	1	3
Basophils (%)	0	0	0	0
Platelets (x10^3^/uL)	428	406	542	566

*Numerical results for standard hematological tests were obtained from the EDTA-anticoagulated blood with an automated Sysmex XN-10 series hematology analyzer (Sysmex America, Inc., Lincolnshire, IL). A 200 leukocyte differential count was obtained by manual blood film review and conversion to absolute numbers. RBC, red blood cell count; MCV, mean cell volume; MCH, mean cell hemoglobin; MCHC, mean cell hemoglobin concentration; WBC, white blood cell count*.

Immunocytochemical staining for CD3 was conducted at the Histology Laboratory of the University of Florida College of Veterinary Medicine to aid in the differentiation of the unique mononuclear cells as monocytes or lymphocytes. A rabbit polyclonal anti-human CD3 antibody (CD3Σ chain; RB-9039-P0, LabVision, Fremont, CA) with cross-reactivity across mammalian and non-mammalian species was applied to wedge-type direct blood films from all four study animals as previously described (22, 23). The slides were counterstained with Harris Hematoxylin (Fisher Scientific, Pittsburgh, PA, USA) and cover slipped. The above procedure was followed to obtain negative controls, except that primary antibody was not applied to the blood films. For positive controls, domestic dog blood films were used. Positive and negative controls were run concurrently with each set of processed slides.

For TEM, a tube of EDTA-anticoagulated blood was centrifuged at 2,500 rpm for 20 min, the plasma was removed, and the remaining buffy coat was preserved by replacing the plasma volume with 2.5% glutaraldehyde in 0.1M phosphate buffer (Electron Microscopy Services, Hatfield, PA 19440). The samples were placed vertically in a refrigerator (4°C) for 24 h, undisturbed. They were then post-fixed in 1% osmium tetroxide, dehydrated through a graded ethanol series (16) and propylene oxide, followed by embedding (EMBED 812, Epon 812 substitute, Electron Microscopy Sciences, Hatfield, PA). Ultrathin sections were prepared with an ultramicrotome (EM UC6, Leica Microsystems, Inc., Buffalo Grove, IL), counterstained with uranyl acetate and lead citrate, and imaged on a transmission electron microscope (Hitachi 7600, Hitachi High Technologies America, Inc., Clarksburg, MD) at the Electron Microscopy Core Facility, University of Florida College of Medicine. Identification of blood cells by TEM was based on nuclear morphology, e.g., non-lobulated or lobulated, and cytoplasmic features, e.g., the relative number, size, shape, and distribution of granules and organelles.

## Results

**Figure 2:**
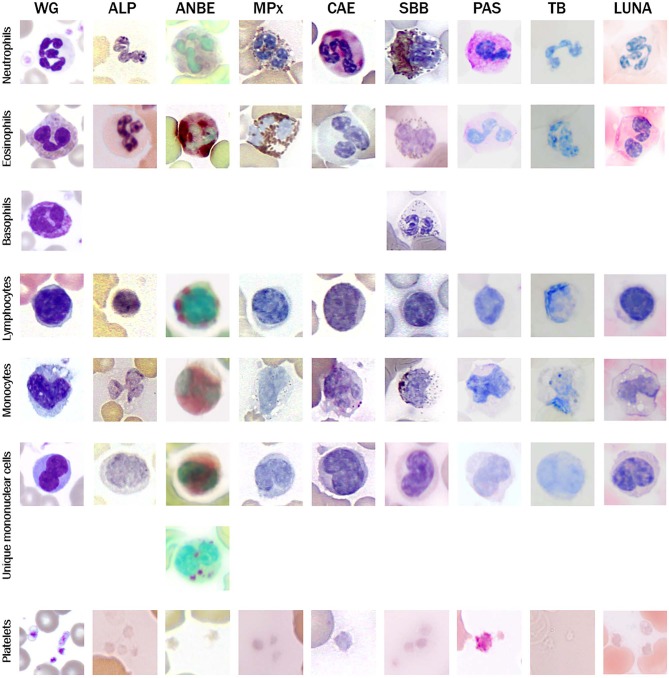
Image composite of representative morphological features of giant panda (*Ailuropoda melanoleuca*) leukocytes in Wright-Giemsa (WG)-stained blood films and blood films stained with eight cytochemical stains (ALP, alkaline phosphatase; ANBE, α-naphthyl butyrate esterase; MPx, myeloperoxidase; CAE, chloroacetate esterase; SBB, Sudan black B; PAS, Periodic-Acid-Schiff; TB, Toluidine blue (pH 2.5-3.0). Basophils were not identified with most of the cytochemical stains.

The blood cells lacked overt morphological abnormalities and hemoparasites were not identified in blood films from all 4 bears. Typical granulocytes (i.e., neutrophils, eosinophils, basophils), mononuclear cells (i.e., lymphocytes, monocytes), and platelets were identified in blood films stained with Wright-Giemsa (Figure 2). In addition, a mononuclear cell with overlapping features of lymphocytes and monocytes, having a moderately indented to occasionally bilobed nucleus was identified in low numbers in the blood film from each bear (termed “unique” mononuclear cell; see below for more detail). Cytochemical staining revealed consistent patterns in leukocytes and platelets, which are described in detail below and summarized (Table 2). Neutrophils, lymphocytes, monocytes, and platelets were identified on TEM; eosinophils and basophils were not identified on TEM, given their low concentrations in the blood of clinically normal giant pandas of this study.

**Table 2:** Cytochemical staining patterns of giant panda (*Ailuropoda melanoleuca*) leukocytes and platelets.

***n* = 4**	**ALP**	**ANBE**	**MPx**	**CAE**	**SBB**	**PAS**	**TB**	**Luna**
Neutrophils	-	C: +/– to +++; some –	C (granular): +++	C (diffuse to chunky multifocal): ++ to +++, rare + focal	C (granular): +++	C (granular): +++	–	–
Eosinophils	-	C: + to +++ (outlining granules)	C (granules): +++	–	C (granular): ++	C: + (cytoplasmic to granular: granules negative)	–	C (grainy, diffuse, outlining granules): +
Basophils	N/A	N/A	N/A	N/A	N/A	N/A	N/A	N/A
Lymphocytes	-	C (multifocal to focal granular): +++; some small and rare large lymphocytes: –	–	–	–	–	–	–
Monocytes	-	C (diffuse): + to ++	C (few granules): +/–	Mostly negative, a few cells in some pandas (1+ granular)	C (few granules): +/–	C (granules): +/–	–	–
Unique mononuclear cells	-	Variable: C either diffuse + to ++ or multifocal to focal granular +++	–	Mostly negative, rare cells in some pandas (1+ granular)	–	–	–	–
Platelets	-	–	–	–	–	C (diffuse to granular): + to ++	–	–

*Staining was subjectively scored by two authors (NS, TS) as negative (–), equivocal (negative or weakly positive, +/–), and weakly (1+), moderately (2+), or strongly (3+) positive with results consolidated between the authors. C, Cytoplasm; ALP, Alkaline phosphatase; ANBE, Alpha-naphthyl butyrate esterase; MPx, Myeloperoxidase; CAE, Chloroacetate esterase; SBB, Sudan black B; PAS, Periodic Acid Schiff; TB, Toluidine blue*.

### Neutrophils

**Figure 3:**
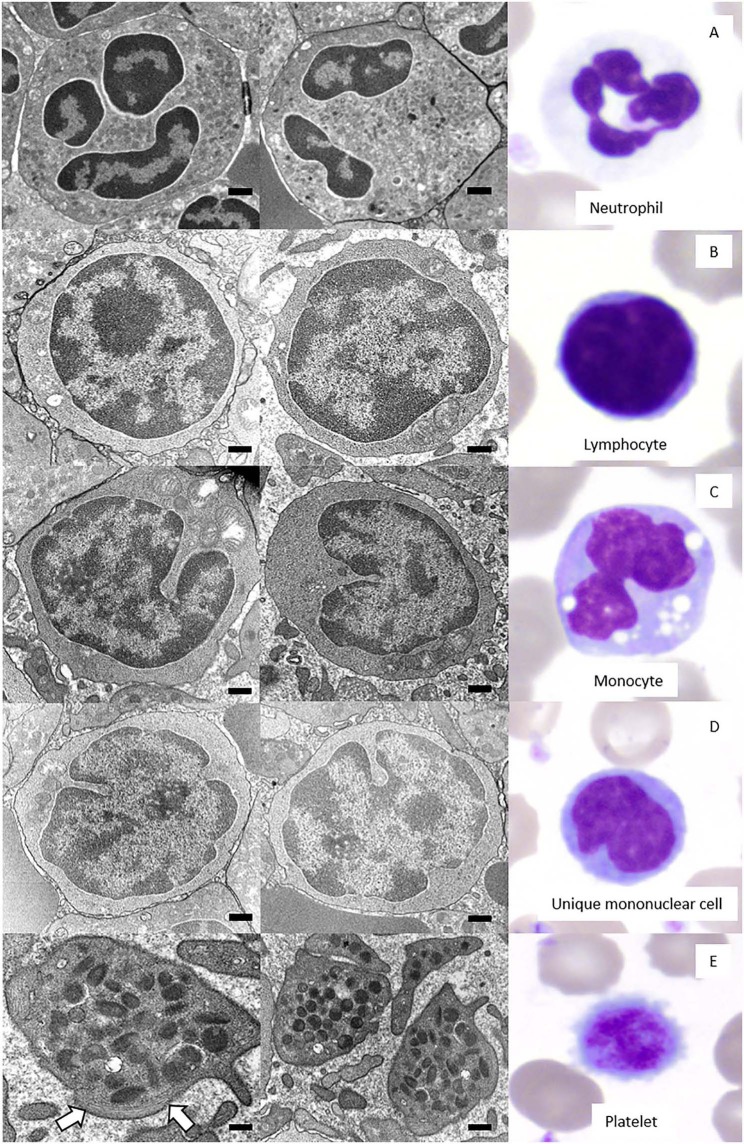
Two-image composite (left and middle panel) of the ultrastructural features of giant panda (*Ailuropoda melanoleuca*) leukocytes and platelets as observed by transmission electron microscopy (TEM) with corresponding Wright-Giemsa-stained images of the relevant cell (right panel). Black size bars = 500 nm for TEM images. **(A)** Neutrophils had a lobulated nucleus predominantly composed of heterochromatin and abundant cytoplasm with numerous granules. **(B)** Lymphocytes had a high nuclear-to-cytoplasmic ratio, a round nucleus with a smoothly curved nuclear membrane, and a thin rim of cytoplasm with a scarcity of organelles, including mitochondria. **(C)** Monocytes had a lower nuclear-to-cytoplasmic ratio compared to lymphocytes, a deeply indented nucleus, and a cytoplasm containing organelles, such as mitochondria. **(D)** Unique mononuclear cells had a nuclear-to-cytoplasmic ratio subjectively between that of lymphocytes and monocytes, with an undulating nuclear membrane that was moderately indented and a cytoplasm containing low numbers of organelles, such as mitochondria. **(E)** Platelets were without a nucleus and had numerous electron-dense cytoplasmic granules and a band of microtubules (arrows) in the peripheral cytoplasm.

Neutrophils were the predominant leukocyte in study animals (Table 1). On Wright-Giemsa-stained blood films, they were ~12–15 μm in diameter with colorless cytoplasm. The nuclei were segmented with clumped chromatin, similar to those in other mammalian species. They showed diffuse cytoplasmic reactivity, ranging from weak to strongly positive for ANBE. They showed strong cytoplasmic staining in a granular pattern for MPx, SBB, and PAS, and moderate to strong, diffuse to chunky multifocal cytoplasmic reactivity for CAE (Figure 2, Table 2). Ultrastructurally, neutrophils were identified by their nuclear lobulation with wide accumulations of heterochromatin along the inner leaflets of the nuclear membrane and relatively less centrally located euchromatin, and their cytoplasm contained primary and secondary granules, as seen in other species (Figure 3A).

### Eosinophils

Eosinophils were slightly larger than neutrophils at ~12–18 μm in diameter and had a segmented nucleus with variable numbers of orange to pink, small round cytoplasmic granules on blood films stained with Wright-Giemsa. They had moderate to strong cytoplasmic positivity for ANBE, which outlined the granules and had stronger staining than monocytes, including chunky deposits. Eosinophil granules were strongly positive for MPx, and moderately positive for SBB (albeit weaker than neutrophils). Eosinophils had weaker granular cytoplasmic positivity for PAS than neutrophils and light patchy grainy cytoplasmic staining for Luna (Figure 2, Table 2). Eosinophils were not definitively identified on TEM.

### Basophils

Rare basophils were identified in Wright-Giemsa-stained blood films (Figure 2). Their morphological features were similar to that observed in other mammalian species with a diameter of 14–16 μm, a segmented nucleus and colorless cytoplasm filled with numerous small round purple granules. No basophils were positively identified using most of the cytochemical stains, making it difficult to determine their cytochemical staining pattern. Two presumptive basophils were positive for SBB (Figure 2) and PAS (not shown), respectively (Table 2). Similarly, basophils were not definitively identified in the examined TEM ultrathin buffy coat sections.

### Lymphocytes

**Figure 4:**
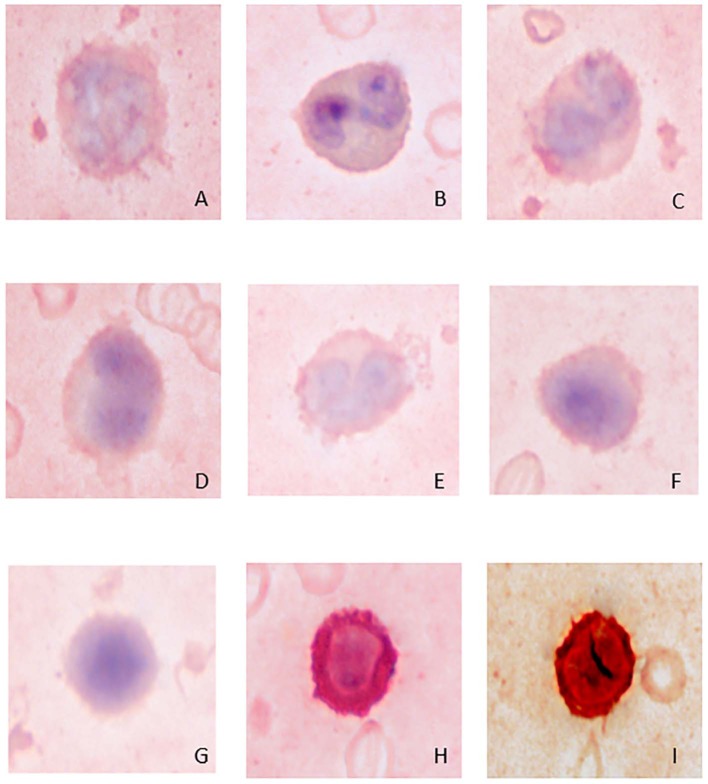
Image composite of immunocytochemical staining reactions for CD3 in giant panda (*Ailuropoda melanoleuca*) leukocytes. **(A)** neutrophil (negative); **(B)** eosinophil (negative); **(C)** monocyte (negative); **(D,E)** unique mononuclear cell (negative); **(F,G)** lymphocytes (negative); **(H)** positive-staining T-lymphocyte; **(I)** canine control showing a positive-staining T-lymphocyte.

Lymphocytes were the second most abundant WBC type in the study animals (Table 1). They were small to large, ranging from ~7–16 μm in diameter, with small well-differentiated lymphocytes being predominant. Lymphocytes were round to oval, typically with a round or minimally clefted nucleus with smooth to clumped chromatin, a high nuclear to cytoplasmic ratio, and a scant to small amount of colorless or light blue cytoplasm. There were rare to low numbers of large and/or reactive lymphocytes with bluer cytoplasm. Rare lymphocytes appeared to have more deeply clefted to lobulated nuclei. On cytochemical staining, small lymphocytes generally showed strong multifocal to focal granular reactivity for ANBE (Figure 2, Table 2). Some small lymphocytes (up to 26%) in 3 animals, and rare large lymphocytes in 1 animal, stained negative for ANBE. Immunocytochemical staining with CD3 showed that most of the giant panda lymphocytes, including rare clefted small lymphocytes, stained positively for CD3 (70–90% of lymphocytes), consistent with T lymphocytes (Figure 4), with low numbers (<15%) being negative for this stain. With TEM, lymphocytes had a round nucleus with a smoothly curved nuclear membrane and a rim of heterochromatin along its inner leaflet, surrounding more centrally located euchromatin. These cells had a high nuclear-to-cytoplasmic ratio and a few cytoplasmic organelles, such as mitochondria (Figure 3B).

### Monocytes

Monocytes ranged from 12 to 18 μm in diameter, had pale blue-grey cytoplasm with a ground glass texture, and a reniform to oval nucleus with lighter less clumped chromatin than lymphocytes on blood films stained with Wright-Giemsa. Some monocytes appeared to have indented to bilobed nuclei. A few monocytes contained low numbers of small clear cytoplasmic vacuoles consistent with reactive monocytes and/or dust-like red-to-pink cytoplasmic granules. On cytochemical staining, monocytes showed diffuse light to moderate cytoplasmic positivity for ANBE. They had variable staining for MPx and SBB, with around 10–20% showing multifocal granular positivity for CAE or PAS (Figure 2, Table 2). Ultrastructurally, monocytes had lower nuclear-to-cytoplasmic ratios compared to lymphocytes, as well as deeply indented nuclear profiles with more cytoplasmic organelles, such as mitochondria, than that seen in lymphocytes. Additionally, there were low numbers of small-diameter membrane-bound vacuoles within the cytoplasm (Figure 3C).

### “Unique” Mononuclear Cells

Low numbers of cells had morphological features that overlapped between lymphocytes and monocytes in Wright-Giemsa-stained blood films. These cells comprised 1–4% of all leukocytes (Table 1). These cells had a nucleus with moderate indentation and variable chromatin features, including smooth or finely stippled chromatin. Several cells had a distinct bilobed nucleus. They ranged in size from 12 to 18 μm in diameter and had a variable amount of pale blue to blue-gray cytoplasm, with a variable nuclear to cytoplasmic ratio. On cytochemical staining, rare cells showed multifocal granular positivity for CAE (Figure 2, Table 2). Although the vast majority (99%) of unique mononuclear cells had lighter diffuse cytoplasmic positivity for ANBE, more consistent with staining characteristics of monocytes, rare (1%) smaller bilobed cells with intense multifocal cytoplasmic staining for ANBE also existed, resembling the staining pattern of lymphocytes (Figure 2). In an attempt to clarify lineage, CD3 immunocytochemical staining was performed since antibodies directed against the zeta chain react across taxa (22–24). We did not perform immunostaining for B cell markers (e.g., CD79a, Pax-5), because available reagents display less species cross-reactivity and require validation prior to use, which was beyond the scope of this study. While rare CD3-positive lymphocytes did have clefted nuclei, most of the cells with moderately indented or bilobed nuclei were negative for CD3, suggesting they were either of monocytic or B cell lineage. The few unique mononuclear cells identified on TEM tended to have a moderately indented nucleus, with a nuclear to cytoplasmic ratio and number of cytoplasmic organelles intermediate between the identified lymphocytes and monocytes of this species. The contour of the nuclear membrane of these cells was undulating, rather than smooth like that of lymphocytes, and less deeply indented than that of monocytes. There were occasional examples of unique mononuclear cells with cytoplasmic organelles aggregated in the area of nuclear indentation, similar to that of the monocytes (Figure 3D). While TEM provided for ultrastructural descriptions of these cells and supported that they were mononuclear cells, the characteristics ascribed to them were based on a small population of cells and did not further differentiate the type of mononuclear cell (i.e., monocytic vs. lymphocytic).

### Platelets

Platelets were negative for ALP, ANBE, CAE, Luna, MPx, SBB, and TB stains. They showed weak to moderate, diffuse to granular cytoplasmic reactivity for PAS (Figure 2), which is typical for platelets across the animal kingdom in mammalian and non-mammalian taxa (15). Rare large platelets were also noted. On TEM, platelets were ovoid to elongated and non-nucleated. Their cytoplasm contained numerous electron-dense, membrane-bound structures consistent with dense secretory granules, i.e. dense bodies, which were largely located in a wide central zone of the cytoplasm, while segments of a circumferential bundle of microtubules were located in the periphery of the cytoplasm (Figure 3), as expected in this cell type (25–27).

## Discussion

This report is the first to characterize normal leukocyte and platelet morphology using routine light microscopy, cytochemistry, immunocytochemistry, and electron microscopy in healthy giant pandas, establishing a baseline for these cell types in this species. With cytochemical staining, reaction patterns seen in other species cannot be directly extrapolated to giant pandas, due to inherent species differences. For example, eosinophils in domestic cats do not normally stain positive with MPx or SBB, while those of other mammals will stain positive for both of these stains (15). In contrast, ultrastructural features of cells are generally conserved across species. By using this combination of routine and specialized modalities, we were able to better characterize the leukocyte lineages of the giant panda. Some leukocytes, such as the unique mononuclear cells noted herein, were potentially misidentified in previous studies that sought to establish hematological ranges for this species. One of these studies utilized the rapid stain, Diff-Quik® (Baxter Healthcare Corp., Dade Division, Miami, Florida 33152, USA), which is inferior to Wright-Giemsa in staining quality with the potential for missing subtle cellular features and staining characteristics (13). The other study derived reference ranges for the different leukocytes using an automated analyzer only, without manual blood film review for morphological assessment and verification of results (14). Additionally, compared to light microscopic blood film review, an automated analyzer likely cannot reliably differentiate unique or different cells found in a given species, such as the unique mononuclear cells identified in the giant pandas of this study.

Leukocyte morphological features were similar for all four healthy giant pandas, both juveniles and adults, in the Wright-Giemsa-stained blood films. Neutrophils were the predominant leukocyte type and basophils were rare, consistent with humans and other members of the order Carnivora (28, 29). Since all giant pandas remained calm and unreactive during voluntary and conditioned phlebotomy, the predominance of neutrophils is unlikely to be related to a stress response. The low number of circulating basophils in the giant pandas of our study is consistent with previous studies in the giant panda and Asiatic black bear (13, 14, 17). Wright-Giemsa also revealed rare large or reactive lymphocytes, rare reactive monocytes, and rare large platelets in both juvenile and adult giant pandas, indicating that rare numbers of antigenically stimulated mononuclear cells can be expected in healthy pandas (25).

Neutrophils had the typical cytochemical reactions seen in most animals, with positive reactions for SBB, CAE, MPx and PAS (15). The results for PAS and SBB are similar to that reported for the Asiatic black bear (17). MPx is the standard stain used to identify neutrophils, while SBB stains lipids in neutrophils. The CAE stain is more specific but less sensitive than MPx or SBB in neutrophils of other mammalian species (15, 30) and was strongly positive in the giant panda neutrophils. Giant panda neutrophils did have a notable difference in cytochemical staining compared to most other mammals, however. They had positive diffuse cytoplasmic reactivity when stained with a substrate for ANBE (a commonly used non-specific esterase). The few other taxa with non-specific esterase positive neutrophils include equine and caprine species which reportedly showed variable positivity, and Asiatic black bears which had moderate to strong positive staining reactions for alpha-naphthyl acetate esterase (ANAE, another non-specific esterase) (15, 17, 29–31). Unlike neutrophils of humans, primates, rabbits, rats, guinea pigs, horses, and ruminants, mature neutrophils from the giant panda were negative for ALP, similar to those of the dog and cat (15, 32). Ultrastructurally, neutrophils had a granular cytoplasm similar to that expected in other mammalian species.

Eosinophils were readily identified on Wright-Giemsa-stained blood films, but were not always easy to differentiate from neutrophils or monocytes in the cytochemical stains that lacked staining in the granules. As expected, they were the only leukocyte to stain positively with the Luna stain, an eosinophil-specific stain (15). Overall, giant panda eosinophils had similar cytochemical staining reactions to other mammals with positive reactions for SBB and MPx, and negative reactions for CAE (15). They shared some cytochemical similarities with the Asiatic black bear (positive for SBB and non-specific esterase) and elephant (positive for MPx and ANBE, negative for ALP and CAE negative), but unlike the Asiatic black bear, eosinophils of the giant panda were PAS positive (17, 33). The ultrastructural features of eosinophils are expected to be similar to that of other species (34), but since none were observed by TEM due to their low number in this study, this assumption cannot be verified.

Lymphocytes and monocytes of giant pandas were morphologically and cytochemically similar to those of other animals. Most of the small lymphocytes of the giant panda showed focal to multifocal strong granular reactivity for non-specific esterase (ANBE used in this study) and lacked reactivity to the other cytochemical stains, similar to that in other species, including the Asiatic black bear (15, 17). This pattern of ANBE positivity has been associated with lymphocytes of T cell origin in other studies (35–38). Interestingly, up to 26% of the small lymphocytes and rare large lymphocytes were ANBE negative. This could be due to weak staining, the presence of only a few small granules, which could be readily missed on cursory review, or non-T cell lineage cells. In contrast, monocytes had diffuse light to moderate cytoplasmic positivity for ANBE, suggesting that ANBE may help differentiate these two mononuclear cells in the giant panda, as in other species. However, it is acknowledged that the staining patterns are quite subjective and staining intensity will also be influenced by pH and other methodological factors, such as reaction times (15). The reactions noted with the rest of the cytochemical stains in the giant panda monocytes were consistent with monocytes of other species, such as the Asiatic black bear (15, 17). The immunocytochemical staining showed that the antibody against CD3 identified T cells in the study animals, with expected negative reactions in the other cell lineages, including monocytes. Ultrastructurally, monocytes had lower nuclear-to-cytoplasmic ratios, more deeply indented nuclear profiles, and were often noted to have slightly more cytoplasmic organelles than lymphocytes.

The different staining patterns in lymphocytes and monocytes with respect to the ANBE stain were helpful when trying to determine the lineage of the “unique” mononuclear cells discovered in the giant panda. Individual cells had light microscopical and ultrastructural features that were seen in lymphocytes and monocytes, making them difficult to categorize with confidence. Most of these cells had moderately indented nuclei, while a few had a more distinctly bilobed nucleus, the latter of which is a feature also seen in monocytic leukocytes of elephants, hyraxes and manatees (members of *Afrotheria*) (33, 39, 40). Although rare cells had ANBE staining characteristics consistent with T lymphocytes (i.e., multifocal cytoplasmic staining), most of the cells in question displayed ANBE staining characteristics consistent with giant panda monocytes, in addition to rare cells showing multifocal granular CAE positivity (15). Additionally, the results of CD3 immunostaining revealed the vast majority of these cells were negative for this T cell marker, supporting the possibility that these cells may in fact be of monocytic lineage. Although B cell origin is also a consideration and can only be excluded by negative staining with validated antibodies (which was beyond the scope of this study), the ANBE staining characteristics make this cell type less likely to be of B cell origin. Due to their low number in blood, it was difficult to conclusively identify defining ultrastructural features of these cells using TEM. While we suspect the unique cell in question should be classified as a monocyte, we acknowledge the limitations of the tools utilized in this study, and recommend that further testing (e.g., double immunostaining using flow cytometry with molecular lymphocyte and monocyte markers) will be required to definitively prove that these cells are not lymphocytes, e.g., B lymphocytes or non-B non-T lymphocytes; however, reagents for such further testing have not yet been identified or validated for this species. In addition to this limitation, we acknowledge that the number of animals included in this study consists of only 4 individuals, due to limited access to individuals of this high-profile species. However, our number of study animals is comparable to other studies on cytochemical staining and morphological leukocyte characterization of various nondomestic species (17, 41–43).

Platelets of the giant panda had classic morphological features and only showed positive cytochemical staining for PAS. The latter is a consistently positive stain in these cells of most species, including avian and reptilian thrombocytes, the mammalian platelet equivalent (15, 44, 45). The negative reactions for the other cytochemical stains mirror that seen in most other species, with some exceptions (e.g., canine platelets are usually positive for non-specific esterases, including ANBE) (15, 17, 35, 46, 47). Ultrastructurally, giant panda platelets had cytoplasmic electron dense granules similar to those seen in other mammals, including the Asiatic black bear (17), as well as a marginal band of microtubules, like that described in humans and other non-human primates (26).

## Conclusions

The results herein will be a useful resource for the expected normal morphological, cytochemical, and ultrastructural findings of leukocytes in this vulnerable and high profile species, and will provide a basis for more accurate hematological interpretation. This information can be used to help clinicians detect deviations from the norm earlier in the course of disease or in response to environmental or physiological changes, as well as with monitoring trends in captive and wild populations over time. We showed that a polyclonal antibody against the epsilon chain of CD3 yielded the expected positive and negative staining reactions in giant panda leukocytes and would be a useful stain for identifying T lymphocytes in cytological or histological samples from this species. Furthermore, the combined use of Wright-Giemsa, cytochemistry, immunocytochemistry, and (to a lesser degree) TEM allowed for the identification of a unique indented to bilobed mononuclear cell found in low numbers in the giant panda, suspected to be of monocyte cell lineage. While cytochemical staining was helpful in determining the cell lineage of unique mononuclear cells, routine Wright-Giemsa evaluation was generally adequate for differential counting of leukocytes. This study also highlights the importance and clinical value of manual blood film review, as it is superior to automated leukocyte differentials in species with unique leukocyte types. Without the detailed leukocyte characterization presented herein, these cells may have otherwise remained unnoticed or been misidentified in clinical cases and future studies, as was the case for the bilobed monocytes of elephants, prior to their accurate identification. Future studies are needed to further characterize and identify this cell type, given the limitations of the tools utilized in this study, as well as identify the hematological changes that occur in giant panda leukocytes in response to specific diseases of concern or to physiological or environmental changes. Additionally, studies focused on validating additional molecular markers for leukocyte phenotyping are warranted.

## Data Availability Statement

All datasets generated for this study are included in the article/supplementary material.

## Ethics Statement

The animal study was reviewed and approved by UF IACUC# 201706823. Written informed consent was obtained from the individual(s) for the publication of any potentially identifiable images or data included in this article.

## Author Contributions

SK, primary and corresponding author and primary scientific investigator, performed phlebotomy on giant pandas, prepared samples, and compiled tables for manuscript. Wrote and edited manuscript. NS performed Wright-Giemsa and immunocytochemical (CD3) staining of giant panda leukocytes and platelets, performed detailed light microscopical assessment of Wright-Giemsa-stained blood films and is one of the two authors who evaluated the cytochemical staining reactions, took photomicrographs, compiled image composites and tables for the manuscript, and reviewed and edited manuscript. SF performed and compiled transmission electron microscopy images of giant panda leukocytes and platelets, and reviewed and edited the manuscript. TS oversaw cytochemical staining of blood films and is one of the two authors who evaluated the cytochemical staining reactions in all the pandas, and reviewed and edited the manuscript. All co-authors contributed to manuscript revision, and read and approved the submitted version.

### Conflict of Interest

The authors declare that the research was conducted in the absence of any commercial or financial relationships that could be construed as a potential conflict of interest.

## Abbreviations

TEM: transmission electron microscopy
EDTA: ethylenediaminetetraacetic acid
PVC: polyvinyl chloride
ALP: alkaline phosphatase
ANBE: α-naphthyl butyrate esterase
MPx: myeloperoxidase
SBB: sudan black
CAE: chloroacetate esterase
PAS: Periodic-Acid-Schiff
TB: Toluidine blue.
